# Review of Modeling Techniques for Analysis and Assessment of RC Beam–Column Joints Subjected to Seismic Loads

**DOI:** 10.3390/ma15217448

**Published:** 2022-10-24

**Authors:** Muhammad Ilyas, Awais Ahmad, Abdullah Riaz, Fayaz Ahmad Khan, Sadaf Sher, Muhammad Waseem, Syeda Zunaira Ali, Yasir Irfan Badrashi, Hafiz Ahmed Waqas, Hermann Seitz, Khan Shahzada, Megersa Kebede Leta

**Affiliations:** 1 Department of Civil Engineering, University of Engineering and Technology, Peshawar 25120, Pakistan; 2 Department of Civil Engineering, Ghulam Ishaq Khan Institute of Engineering Sciences and Technology, Topi 23640, Pakistan; 3 Faculty of Mechanical Engineering and Marine Technology, University of Rostock, Justus-von-Liebig-Weg 6, 18059 Rostock, Germany; 4 National Institute of Urban Infrastructure Planning, Hayatabad, Peshawar 25120, Pakistan; 5 U.S. Pakistan Center for Advanced Studies in Water, Mehran University of Engineering & Technology, Jamshoro 76062, Pakistan; 6 Department of Life, Light and Matter, University of Rostock, Albert Einstein-Str. 25, 18059 Rostock, Germany; 7 Faculty of Agriculture and Environmental Sciences, University of Rostock, 18059 Rostock, Germany

**Keywords:** bar-slip, beam–column joints, interface shear, multi-spring macro-models, panel shear, pinching, zero-length elements

## Abstract

Beam–column connections are the most critical components of reinforced concrete (RC) structures. They serve as a load transfer path and take a significant portion of the overall shear. Joints in RC structures constructed with no seismic provisions have an insufficient capacity and ductility under lateral loading and can cause the progressive failure of the entire structure. The joint may fail in the shear prior to the connecting beam and column elements. Therefore, several modeling techniques have been devised in the past to capture the non-linear response of such joints. Modeling techniques used to capture the non-linear response of reinforced-concrete-beam–column joints range from simplified lumped plasticity models to detailed fiber-based finite element (FE) models. The macro-modeling technique for joint modeling is highly efficient in terms of the computational effort, analysis time, and computer memory requirements, and is one of the most widely used modeling techniques. The non-linear shear response of the joint panel and interface bond–slip mechanism are concentrated in zero-length linear and rotational springs while the connecting elements are modeled through elastic elements. The shear response of joint panels has also been captured through rigid panel boundary elements with rotational springs. The computational efficiency of these models is significantly high compared to continuum models, as each joint act as a separate supe-element. This paper aims to provide an up-to-date review of macro-modeling techniques for the analysis and assessment of RC-beam–column connections subjected to lateral loads. A thorough understanding of existing models is necessary for developing new mechanically adequate and computationally efficient joint models for the analysis and assessment of deficient RC connections. This paper will provide a basis for further research on the topic and will assist in the modification and optimization of existing models. As each model is critically evaluated, and their respective capabilities and limitations are explored, it should help researchers to improve and build on modeling techniques both in terms of accuracy and computational efficiency.

## 1. Introduction

In fully code-compliant ductile frames, the beam–column joints exhibit two main mechanisms of shear transfer: the diagonal truss mechanism and the boundary forces producing tensile effects in the joint core. Transverse reinforcement provides confinement to the core and strengthens the compression strut even after the spalling of the concrete cover [1]. When the steel concrete bond is strong and a sufficient anchorage length is provided, the shear demand on the joint increases, which requires a large amount of transverse reinforcement [2]. On the contrary, when the bond condition is poor, rigid joint rotation occurs to accommodate large slippage, which cannot be controlled even after providing additional transverse reinforcement [1]. In order to evaluate the joint shear capacity and strength reduction due to design and construction deficiencies, capable analytical and numerical models are necessary to simulate the shear deformation and bond slip mechanism, affecting the seismic behavior of joints.

**Figure 1:**
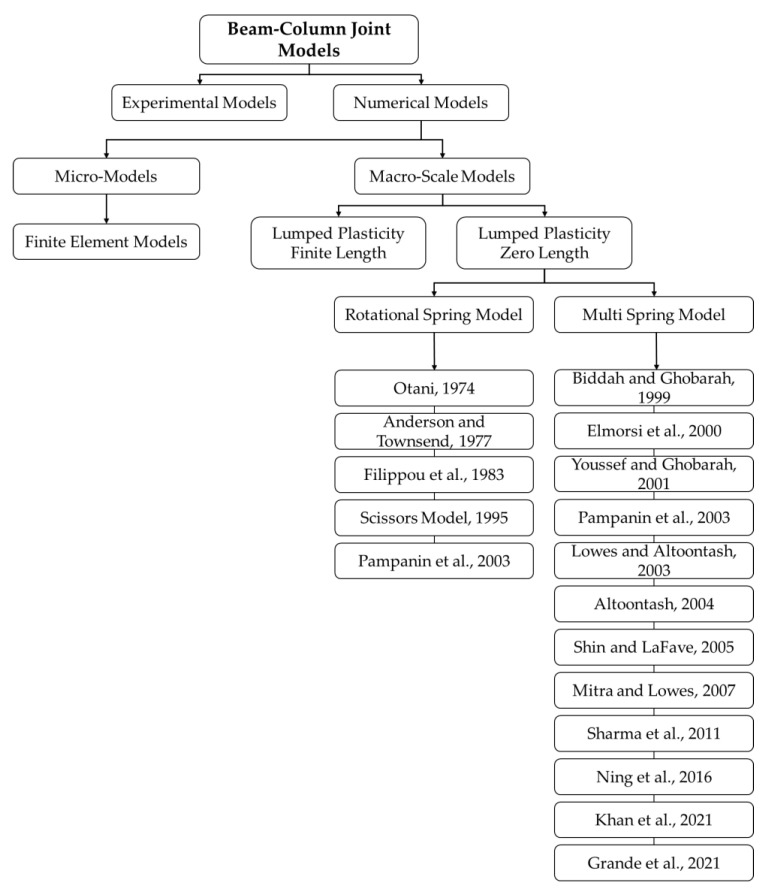
Classification of common models used for analysis and assessment of RC joints.

The development of new optimized modeling approaches used to simulate the behavior of RC joints is an active research topic. Significant research has been conducted on the topic and various modeling approaches have been proposed in the last few decades [1,3,4,5,6]. Literature reveals that the main mechanisms governing the RC joint response are panel shear deformation and the interface bond slip mechanism [7]. Existing beam–column joint models can generally be classified as experimental models and analytical models as shown in Figure 1.

### 1.1. Research Impact

In RC structures, when the beam–column joints are modeled as elastic, the shear strength is highly overestimated. Joints are to be modeled with non-linear continuum or lumped plasticity models for a better and accurate approximation of response. Continuum models require high computational effort and expensive hardware. On the other hand, lumped plasticity models require minimum computational effort with a significantly accurate response prediction. Therefore, effort was made in this paper to critically review the existing lumped plasticity, multi-spring beam–column joint models, which will significantly contribute to the understanding of joint mechanics and modeling approaches. For the development of new optimized models, the understanding of previous models is crucial. Multiple authors have added literature reviews regarding specific joint models that they have modified or employed in their research. No review paper exists in the field that provides a sequential detailed overview and critical analysis of RC joint models. Merits and limitations of each model are discussed in terms of the accuracy and computational effort.

### 1.2. Experimental Models

Some of the earlier studies introduced ‘plastic hinges’ between the connected elastic beam–column elements to simulate the non-linear behavior of RC frames. Such studies were generally based on experimental investigations of full or reduced-scale beam–column sub-assemblages tested under a monotonic or cyclic load. For the modeling of the inelastic response under a cyclic load, various hysteretic rules were defined to study different behavioral phases under multiple loading and unloading cycles and subsequent strength and stiffness degradation [8].

Townsend and Hanson [9] devised a series of formulations to capture the cyclic behavior of beam–column connections and explain the strength and stiffness deterioration in subsequent cycles. The combination of several formulations for the first quarter cycle yields the following governing equation:

$$\frac{M}{M_{y}}=0.172+1.03\gamma -0.167\gamma^{2}-0.00846\gamma^{3}$$

where γ is the hinge rotation and the derivative of *M*/*M_y_* gives joint stiffness.

Anderson and Townsend [10] proposed a degrading trilinear joint model based on a set of experimental results from an external beam–column joint. However, these models considered concentrated plasticity at the member ends, which overestimated the curvature and story drift. To overcome this limitation, Soleimani et al. [11] introduced the concept of an effective length, which is derived by multiplying the curvature at the beam–column interface by the fixed end rotation. The parameters characterizing the connection’s hysteretic behavior were chosen based on experimental observations without any consideration for joint mechanics. As a result, the application of such models for joints in other orientations and loading circumstances is dubious.

### 1.3. Analytical Models

Analytical models are bifurcated as implicit and explicit models. In implicit models, plastic hinges or springs are installed at the member ends to record the strength and stiffness loss caused by joint deterioration [8]. However, with implicit models, it is difficult to estimate shear deformation. In explicit models, the nonlinear response of the joint is characterized by joining the centroids of connecting elements with finite volume macro-elements. Using these models, joint kinematics is satisfied, and joint elements are easily calibrated. However, the analysis of several rotational springs may cause convergence issues and numerical instability when simulating the nonlinear response of an entire frame. Various researchers have proposed several models based on analytical studies to capture the response of RC joints. These are classified as rotational hinge models and multi-spring models based on the number of non-linear springs or hinges used to predict shear and bar-slip phenomena.

#### 1.3.1. Rotational Hinge Models

Typical input parameters for rotational hinge models are a stress–strain envelope and hysteretic rules specifying cyclic behavior. These models are mostly controlled and directed by a multilinear monotonic curve, the key points of which are defined by multiple constitutive models based on empirical equations and experimental observations. The cyclic behavior is controlled by several calibration parameters corresponding to the required level of the pinching effect, strength, stiffness, and energy degradation in subsequent cycles based on the actual structural response. The earliest model in this domain was developed by Giberson [12] and was based on the idea that the joint should be allowed to deform plastically during lateral loads. The non-linear response due to the shear demand on the beam coming from the flexural response of the connecting elements was captured through two rotational hinges positioned at the member ends.

Otani [10,13] initiated the idea of introducing discrete inelastic action to analyze the non-linear behavior of joints in RC frames. They tried to capture the inelastic flexural response of frames using plastic hinges at the joint location. Otani [13] defined the key phases of the response envelop based on bilinear idealization. The embedment length was assumed to be enough for exhausting the full steel capacity and it was assumed that stresses were uniform throughout its length. It was observed that the fixed end rotation is in proportion to the square of the interface moment. For the cyclic response, the Takeda hysteretic rule [14] was employed. The pinching of the hysteretic response due to the bond–slip mechanism and interface shear was incorporated in approximations by Banon et al. [15]. In these models, there is no adequate mechanism for capturing the inelastic response at the interface, whereas the panel shear distortion and corresponding stress response is captured with significant precision [13,14,16]. Anderson and Townsend [10] suggested two degrading trilinear models, out of which, one considers the connection effect. Banon et al. [15] used a bilinear response curve alongside the Takeda hysteretic rule [14], making the same assumptions as Otani [10]. They also considered the pinching effect caused by the panel shear and bond–slip mechanism. The model captures inelastic deformations caused by reinforcement slippage. However, these models underestimate the joint strength and stiffness, and the mechanism causing stiffness degradation is not analytically explained. The models create an interaction between the column ends, and the derivation of moment–rotation (M-θ) relationships is not unique but dependent on both ends. Furthermore, they all share the limitation of not accounting for the bar–slip mechanism in interior joints [17].

**Figure 2:**
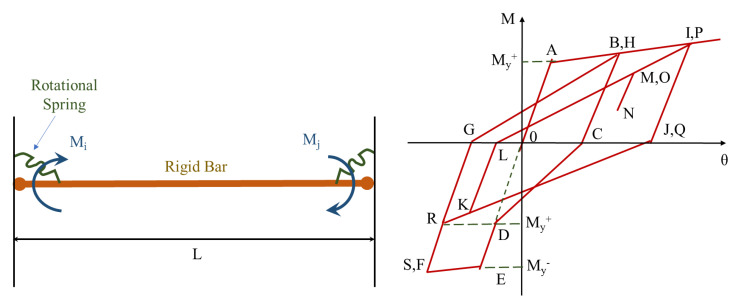
Fixed-end rotations and corresponding deterioration in joint hysteretic behavior, reproduced from literature [19].

There are many alternative approaches used in the literature for the non-linear response approximation of the joint shear response; however, such models fail to adequately account for the joint shear behavior, though sufficiently consider the flexural demands coming from the connecting beams and column [18,19]. Filippou et al. [18,19] provided a model that considers the influence of flexural reinforcement debonding on joint hysteretic behavior. The joint model, shown in Figure 2, compensates for fixed-end rotations at the joint interface because of debonding and reinforcement slippage in the joint. The model consists of a discrete rotational spring at each end joined by a rigid beam element. The rotational springs’ M-θ relationship is calculated using the model by Filippou et al. [19], which accounts for the shape, material properties, and reinforcement scheme of the connection. The following equation was proposed to estimate the decreased stiffness beyond the yield point:

$$K_{sp}=\frac{M_{u}-M_{y}}{\vartheta_{pl}}$$

At each link, a unique M-θ curve can be defined, which is based on a bilinear elastic-strain-hardening relationship [20]. The plastic zone length is given by

$$z_{c}=\frac{M-M_{y}}{V}$$

where *M* is the real-time value of the bending moment and *V* is the real-time value of the shear force.

The model is easy to use, significantly accurate, and solely based on joint mechanics. However, the model fails to simulate panel shear and diagonal cracking under cyclic loads.

**Figure 3:**
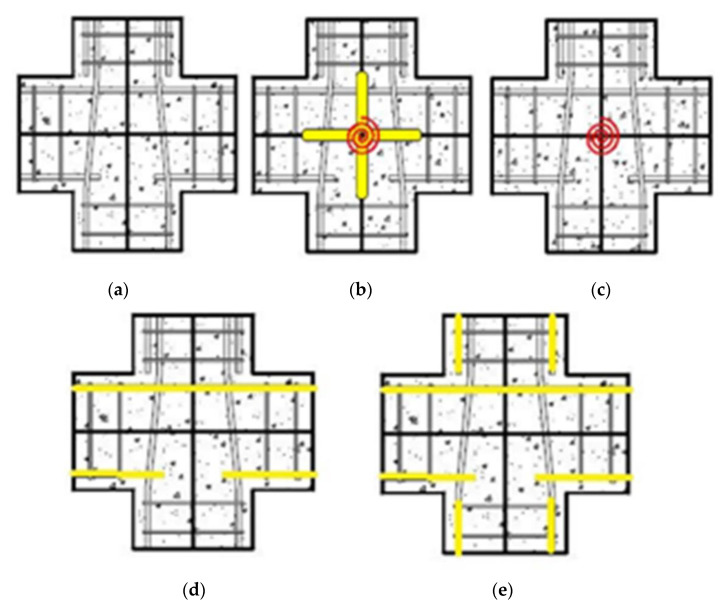
(**a**) Joint centerline model, (**b**,**c**) scissors model with and without rigid links, (**d**) centerline model with decreased beam-bending capacity, and (**e**) centerline model with decreased bending capacities of both beams and columns, reproduced from literature [21].

Hoffmann et al. [21] introduced a model to account for detailing deficiencies by reducing moment capacities of adjacent beams and columns to develop both a panel shear and bar–slip mechanism as given by the below equation and shown in Figure 3.

$$A_{eff}=\frac{l_{emb}}{l_{db}}*A_{s}$$

$$M_{pullout}=\frac{l_{emb}}{l_{db}}*M_{y}\;$$

where *l_emb_* is the embedment length, *l_db_* is the development length, and *A_s_* is the steel area.

The modified Park model [22] was used for damage analysis, which estimates damage in terms of deformation and energy dissipation. The overall damage is calculated by superimposing deformation and hysteretic damage.

$$D=\frac{\delta_{m}}{\delta_{u}}+\frac{\beta }{\delta_{u}P_{y}}\int dE$$

where *D* is the damage index (0–1), $\delta_{m}$ is the maximum deflection, $\delta_{u}$ is the ultimate deformation, β is the strength deterioration rate, $P_{y}$ is the yield capacity, and ∫dE represents hysteretic energy dissipation.

**Figure 4:**
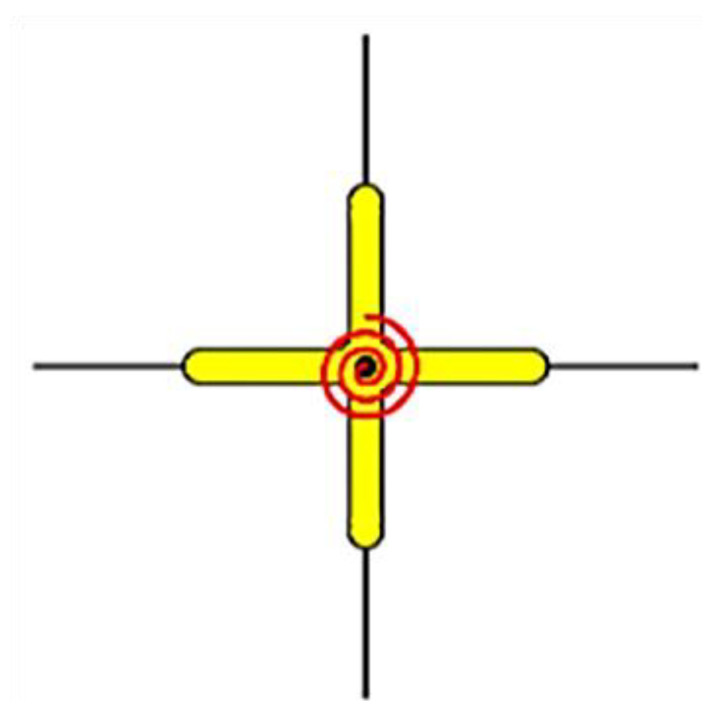
Alath and Kunnath model with central rotational spring and rigid offsets, reproduced from literature [3].

The above modeling approaches fail to account for joint kinematics as they cannot explicitly model the deformations that represent the finite region. These approaches are limited in the response prediction of the finite length of the joint panel. The flexural rigidity of the joint was not considered in joint mechanics. To characterize joint kinematics, Alath and Kunnath [3] used rigid connections to account for the joint’s flexural rigidity and its finite size. A rotational spring with a deteriorating hysteresis loop was used to simulate joint panel shear deformation as shown in Figure 4. The individual rotations of connecting elements were represented by their respective constitutive models and hysteresis rules. The shear stress–strain behavior was empirically derived, whereas the cyclic hysteretic response was based on experimental results. However, there is no non-linear response prediction mechanism in the joint, except for a rotational spring representing the shear behavior of the concrete core. Moreover, this model fails to predict the interface shear or bond–slip mechanism.

Several studies have attempted to reduce modelling uncertainties and computational effort due to the interface shear and bond–slip mechanism by introducing a zero-length rotational spring to simulate an inelastic response of the beam–column connection [3,23,24]. El-Metwally and Chen [23] modeled the joint as a discrete rotational hinge using the principles of thermodynamics to determine stiffness. The model is based on two assumptions: the cyclic nonlinear response is controlled by the development length of longitudinal reinforcement; energy dissipation, because of bond deterioration, is constant for all joints. The input parameters are highly dependent on the M-θ relationship, which is a limitation of the model. Kunnath et al. [25] modified the moment capabilities of the connecting elements in pre-1970 frames to implicitly model the anchorage deficiency and joint shear capacity. The debonding capacity of the beam flexural reinforcement was estimated by multiplying the ratio of the embedded bar to the required development length by the section yield moment (*M_y_*). In the case of discontinuous reinforcement, the yield strength (*f_y_*) had to be decreased by the ratio of the provided to required development length. The moment capacities of the connecting elements were decreased to achieve shear failure. The nonlinear dynamic analysis of the proposed model in multi-story deficient RC frames revealed vulnerability to shear failure and soft-story effects.

**Figure 5:**
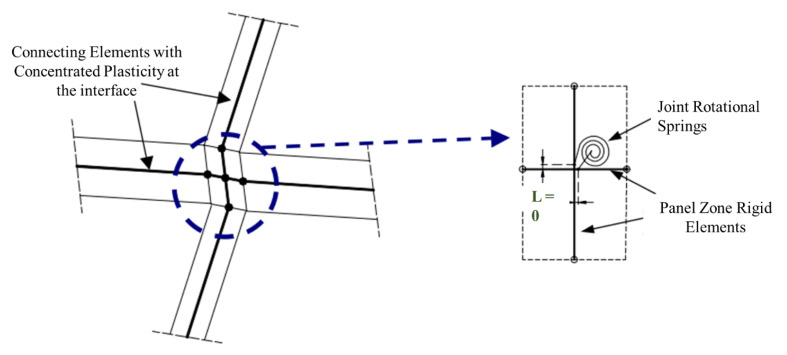
Nonlinear response characterization of rotational springs using a targeted plasticity technique, reproduced from literature [26].

Pampanin et al. [26] proposed that a plastic hinge can be replaced with a shear hinge corresponding to joint degradation. Both the linear and nonlinear response can be characterized using a targeted plasticity technique, where a rotational spring used for a relative rotation of the connecting elements is provided as shown in Figure 5. The monotonic M-θ properties for springs can be calculated from equilibrium equations of the adjacent elements, which correspond to key tensile stress levels in the joint panel zone’s mid-depth. The Ruaumoko Carr [27] finite element programming code was used for modeling and analysis. The cyclic behavior is represented by a modified Stewart hysteresis rule that incorporates the “pinching” effect caused by the reinforcement slip and joint shear cracking [28]. The model fails to account for strength degradation upon diagonal cracking. The hysteretic pinching behavior in subsequent cycles is also not considered. Furthermore, the model does not account for the bar–slip mechanism; however, it considers it indirectly through shear simulation.

Wang et al. [29] developed a model for the shear response approximation of RC beam–column connections subjected to lateral loads based on theoretical considerations. The authors modeled the joint panel reinforced concrete as homogeneous and accounted for the transverse reinforcement through the nominal tensile strength of concrete. Yu and Tan [30] proposed a component-based connection model and incorporated it into a macro-model-based numerical framework where connecting elements were modeled with deformation-based fiber elements. The joint model comprises multiple springs to capture the bond–slip mechanism when exposed to huge tension.

**Figure 6:**
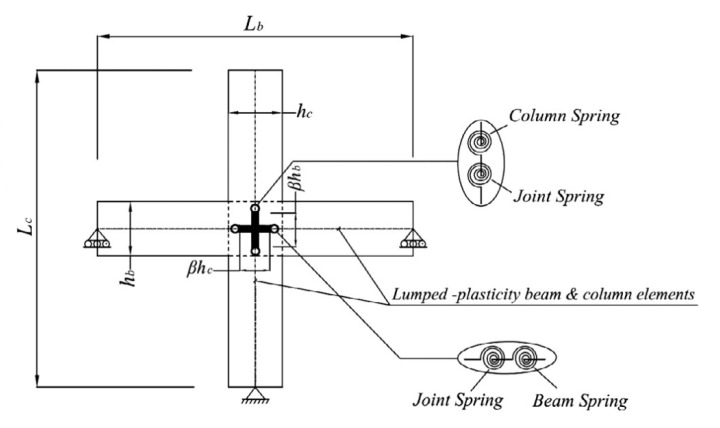
The proposed beam–column joint element with rigid offsets and details of rotational springs, based on literature [31].

Omidi and Behnamfar [31] proposed a simplified rotational spring model for the non-linear cyclic response approximation of RC beam–column joints. The joint model was set to have a concentrated plasticity with rigid offsets components as shown in Figure 6. The rigid offsets were calibrated with a shear–demand ratio producing a good approximation of the initial stiffness of the joint. Two springs at the end of each connecting member were provided in series. The non-linear response of both the connecting element and the joint was captured through these two springs at the end of each member. All of the rotational springs were defined by a separate M-θ relationship. Each one of the rotational springs possessed its own moment–rotation response curve. The model-simulated response very closely confirms the experimental observations of RC joints. However, the study included only the internal joints in which the confinement effects of the transverse beams play a major role as opposed to exterior or corner joints. The study also did not account for the bond mechanism in the joint.

The load–drift curves derived from the assumed rigid offsets were compared to the experimental database and those suggested by FEMA356 and ASCE/SEI 41-06. The initial stiffness was quite closely captured, with a discrepancy of 20.3% for FEMA356 and 5.4% for ASCE/SEI 41-06.

##### Summary of Rotational Spring Models

With only a minor increase in the computational cost, the rotational hinge joint models allow for an independent characterization of the inelastic joint response, which is an easy and more reliable way to replace the conventional practice of modeling the joints as rigid elastic elements. However, this modeling technique complicates design objectives and precise calibration techniques regarding diverse orientations and loading scenarios. It necessitates the utilization of extensive experimental data to construct an M-θ curve. Developing a model for simulating the joint response with various design features necessitates either a complex calibration technique with enormous data sets or multiple joint models with diverse design details. The models’ applications are limited as experimental data of all possible orientations and loading scenarios are not available for calibration. The M-θ curves can be developed by using the constitutive models proposed by various researchers in the past [17], [32,33,34,35,36,37,38]. The literature constitutive models are in terms of shear stress and strain, which can be converted to M-θ using joint mechanics.

The onset of cracking, shear stress (*τ*_1_), was proposed by by Uzumeri [38].

$$\tau_{1}=0.92\;\sqrt{f_{c}}\sqrt{1+0.29\sigma_{j}}\;$$

The value of maximum shear stress ($\tau_{\max}$) has been proposed by the following researchers in the past.

a. Kim and LaFave [37]
  $$\tau_{\max}=0.483\;{\left(BI\right)}^{0.3}{\left(f_{c}\right)}^{0.75}$$
  $$BI=\frac{A_{s,b}\;f_{y,b}}{b_{b}\cdot h_{b}\cdot f_{c}\;}$$
b. Vollum and Newman [36]
  $$\tau_{\max}=0.642\beta \left[1+0.555\left(2-\frac{h_{b}}{h_{c}}\right)\right]\sqrt{f_{c}}\;$$
c. Jeon [35]
  $$\tau_{\max}=0.409{\left(BI\right)}^{0.495}{\left(f_{c}\right)}^{0.941}\;$$

For the remaining values of pre-peak and post-peak shear stress and strains, the models proposed by various researchers can be utilized [17,32,34,39].

#### 1.3.2. Multi-Spring Models

**Figure 7:**
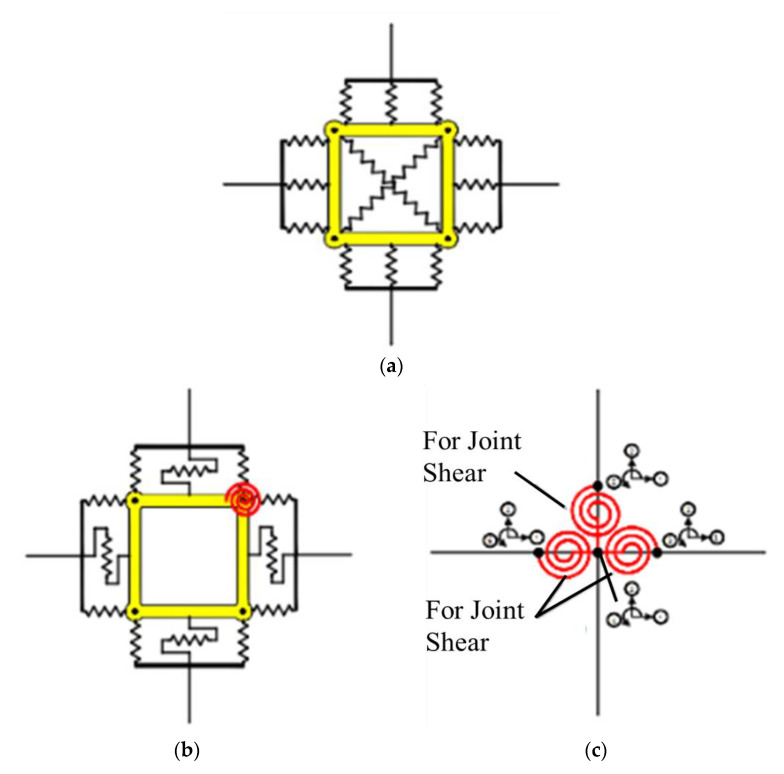

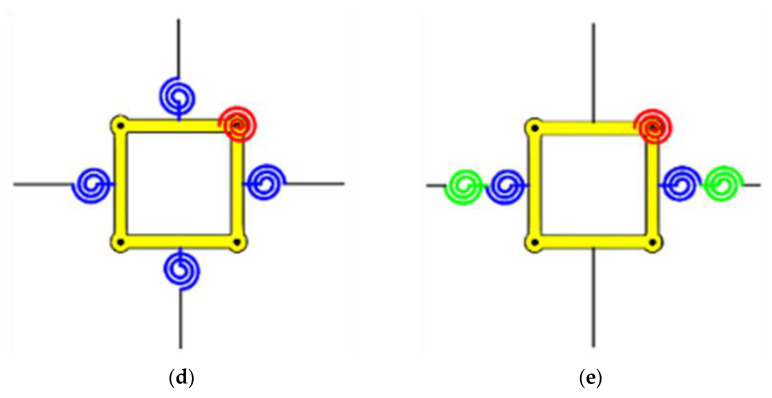
The most common zero-length (**a**) rotational spring model and (**b**–**e**) multi-spring models, based on literature [49].

The rotational spring models have been extended to multi-spring models, which are more realistic and objective. Instead of a partial approximation of the joint shear response by employing a single rotational spring, this method suggests using a combination of zero-length linear or rotational springs to simulate the overall response with individual units. In multi-spring models, the non-linear joint mechanics are captured through zero-length one-dimensional elements [7,34,40,41,42]. Using these models, the joint shear cracking and bond deterioration may be examined separately using separate elements for individual responses [43]. These models can also capture concrete crushing and bond–slip behavior [44]. Damage is estimated by using a lump plasticity approach and the amount of energy dissipated [8,26,45]. The estimated damage can be in terms of the energy or cycle. The cyclic degradation can be computed in terms of strength and stiffness deterioration and the corresponding loop energy variation. Some common multi-spring models are (a) Biddah and Ghobarah [5], (b) Youssef and Ghobarah [46], (c) Lowes and Altoontash [47], (d) Altoontash [48], and (e) Shin and LaFave [40] as shown in Figure 7.

Biddah and Ghobarah [5] modeled the panel shear and interface bond–slip mechanism using two non-linear springs in series as shown in Figure 7a. The shear-force–deformation relationship and shear capacity were determined using softened truss model theory [50], balanced by tensile forces in horizonal and vertical reinforcement. The softening effect due to diagonal cracking caused by tensile stresses is considered in the concrete stress–strain relationship [51]. This macro-model was incorporated in SARCF (non-linear dynamic analysis software) [52]. A bilinear model was used to simulate the bond–slip deformation. A pinching hysteresis relationship was used to capture the cyclic response of bond–slip springs.

Constitutive laws have been defined for various phases, conditions, and material variations; before cracking, the stress and strain has been related as

$$f_{c1}=E_{c}\;\epsilon_{1}$$

After cracking,

$$f_{c1}=\frac{\alpha_{1}\alpha_{2}\;f_{cr}}{1+\sqrt{500\epsilon_{1}}}$$

where, in addition to the symbols discussed above, $f_{cr}=0.33\sqrt{f_{c}\prime }\;\mathrm{MPa}$.

However, the model cannot account for two important mechanisms: the core confinement mechanism due to transverse reinforcement and the contribution of a slab to the joint shear resistance. The authors have referred to possible solutions using experimental results from literature [53,54].

**Figure 8:**
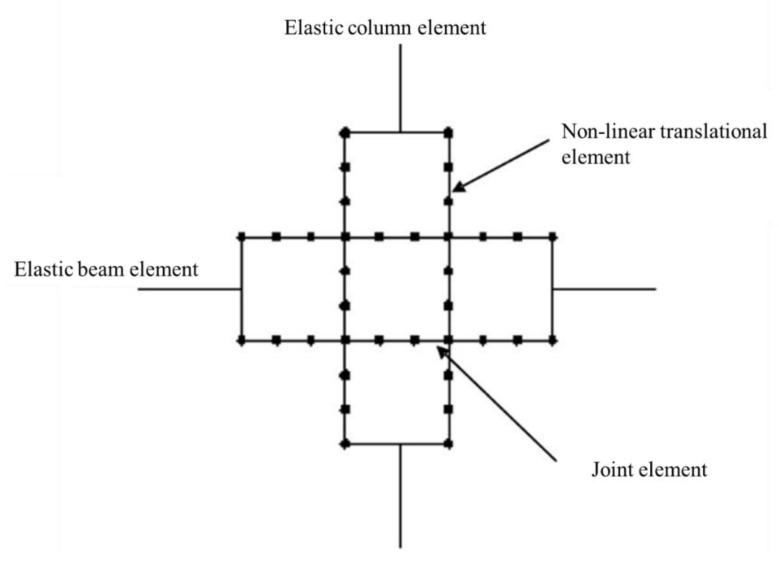
Elastic beams and columns connected to the joint through non-linear translational springs, based on literature [55].

Elmorsi et al. [55] proposed a method in which elastic components are used to characterize beams and columns, and non-linear transitional elements are used to connect them to the joint. Another element, consisting of ten joints, is used to model the effective node panel region as shown in Figure 8. The proposed model accounts for material behavior, as steel and concrete constitutive relationships are provided. The pre- and post-cracking behavior of concrete is determined by two separate relationships. Non-linear pieces are added around the joint panel to simulate longitudinal reinforcing steel bars. To depict reinforcement anchorage loss, a “bond–slip element” is also added to the model. The authors have supplemented experimental and FE results with existing constitutive material models for a better approximation of the response [56,57,58,59]. The monotonic response envelop reduction was computed using correlations proposed by [60,61], whereas the unloading and reloading rules were taken from [62,63]. However, the bond–slip mechanism is simulated locally and its effects on the overall joint behavior cannot be taken into account through the use of this model. The slip mechanism is predicted analytically, and no comparison is made with finite element models.

Youssef and Ghobarah [46] presented a model used to simulate the shear behavior of reinforced-concrete-beam–column joints. The joint panel is modeled through four rigid elements with a pin connection at each corner. The connecting beams and columns are idealized as elastic line elements. The shear and bond–slip mechanisms at the interface are captured through non-linear zero-length elements between elastic beams and columns and rigid joint boundary elements. The elastic beams and columns are connected to the joint through the use of three zero-length springs, simulating bar–slip and interface shear. A shear hinge is provided at each diagonal to simulate joint panel deformation as shown in Figure 7c. The hysteretic rule by Ghobarah and Youssef 1999 [5] was employed to account for stiffness degradation and energy absorption in subsequent cycles. For the prediction of shear deformation, the authors employed the given equation:

$$\gamma =\frac{\gamma_{h1}+\gamma_{h2}}{2}+\frac{\gamma_{v1}+\gamma_{v2}}{2}=\frac{2\Delta D}{D\;\sin\;\left(2\varphi \right)}$$

where both the terms ($\gamma_{h}\;\mathrm{and}\;\gamma_{v}$) give the average shear deformation in the horizontal and vertical direction, respectively, *D* is the original diagonal length, and φ is the angle that the diagonal makes with horizontal plane.

The model can predict pinching, stiffness degradation, and energy absorption with reasonable accuracy. However, this modeling technique is complicated and time-consuming due to the demand of multiple springs and a different rule for each one.

**Figure 9:**
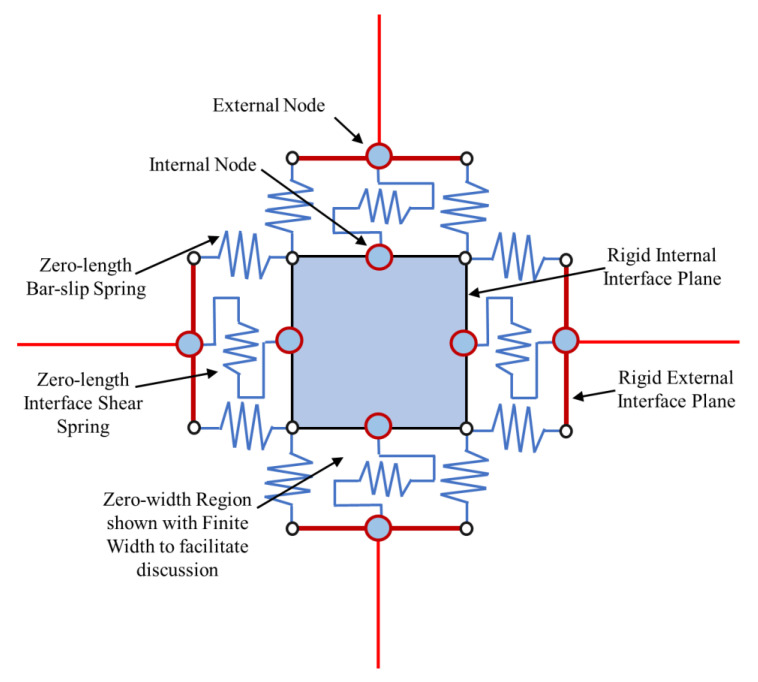
Beam–column joint with zero-length interface shear and bar–slip springs and shear panel, reproduced from literature [47].

**Figure 10:**
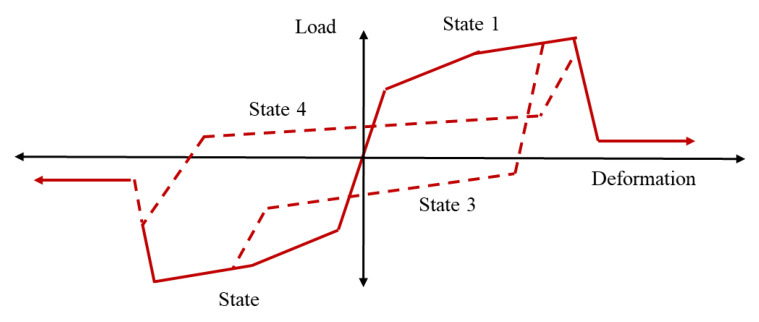
Strength and stiffness deterioration in subsequent cycles, reproduced from literature [47].

Lowes et al. [47] proposed a conventional super element model that represents the basic inelastic mechanics of RC joints. It is made up of thirteen one-dimensional components that give nine more degrees of freedom to the non-linear system. The components of the super-element include a shear-panel, rotational shear springs, and bar–slip springs at the interface as shown in Figure 9. For rotational spring calibration, one-dimensional material models are required, which can characterize the load–deformation relationship as shown in Figure 10. For shear strength estimation and strength and stiffness loss under lateral loads, the modified compression field theory [51] was used. To simulate the non-linear joint panel response under a cyclic load, experimental data were acquired from Stevens et al. [64]. The hysteresis loop obtained was highly pinched. The interface–shear components were considered to have a rather stiff elastic load–deformation response. The authors defined calibration models for each of these responses. By using these calibration models, users can predict the joint shear response as a function of the compressive strength of concrete, yield strength of steel, and dimensions of the joint. A comparison of the models reveals that these are appropriate for simulating the response under the effect of a moderate seismic load demand. It also provided hysteretic rules for joint damage and strength and stiffness deterioration in subsequent cycles as shown in Figure 7. The damage rules proposed in the model are similar to that of Park and Ang, represented in terms of damage index $\delta_{i}$:

$$\delta_{i}=\left(\alpha_{1}{\left({\tilde{d}}_{max}\right)}^{\alpha_{3}}+\alpha_{2}{\left(\frac{E_{i}}{E_{monotonic}}\right)}^{\alpha 4}\right)$$

where ${\tilde{d}}_{max}=\max\left[\frac{d_{max,i}}{def_{max}},\;\frac{d_{min,i}}{def_{min}}\right]\;$ and $E_{i}=\int dE$ over the load history.

However, this modeling technique is complicated and time-consuming due to the demand of multiple springs and, as each spring has its own constitutive and hysteretic rules, its applicability is restricted.

Lowes et al. [65] used experimental data to simulate elastic shear behavior at the interface. Experimental models with at least minimum ties confining the joint core were considered, as the shear is largely carried by a compression strut in joints with minimal quantities of transverse reinforcement, a mechanism that is stronger and stiffer than expected by the MCFT. However, this study eliminated joints with no transverse reinforcement, which is a common feature of GLD RC frames. Altoontash [48] made certain modifications to Lowes and Altoontash’s [47] model in order to make it simple and computationally efficient. The author proposed simulating the interface shear and bond–slip mechanism by using four rotational springs. The joint panel deformation was simulated using a single rotational spring at the center as shown in Figure 7e. The MCFT was utilized to approximate the shear stress–strain relationship, whereas the cyclic behavior was calibrated with an experimental hysteretic response. To simulate the plastic hinge rotation due to the beam elastic behavior and bond–slip mechanism at the interface separately, two rotational springs were employed in series. The behavior of ductile RC beam–column sub-assemblages designed and constructed in compliance with modern seismic specifications was simulated using the given joint model. However, the model cannot simulate the shear response and bond–slip mechanism of RC joints without transverse reinforcement, even after the modifications.

Shin and LaFave [33] simulated the joint panel shear response through a rigid joint boundary element. The interface shear due to the connecting elastic elements is depicted by four rotational hinges as shown in Figure 7f. The shear stress–strain response was estimated using MCFT, whereas the cyclic behavior was calibrated to an experimental hysteretic response. A comparison of joint shear stress level was made for ACI-318-02 and ACI-352R-02 using the below equation.

$$\gamma =\frac{V_{u,j}}{\sqrt{f_{c}\prime }b_{j}h_{c}}$$

where $V_{u,j}$ represents the floating point value for shear force; *b_j_* is the effective joint width; and *h_c_* is the column depth.

To simulate the plastic hinge rotation due to the beam elastic behavior and debonding phenomenon of the reinforcement, two rotational springs were employed in series. The bond–slip constitutive rules employed for the calibration of interface rotation were derived from the authors’ earlier research [33,39,66]. The joint model is to be employed for the analysis and assessment of ductile RC frames that have been built and documented in accordance with recent seismic code criteria. However, the MCFT used in this model is unsuitable as it underestimates the joint shear strength of the joint without shear reinforcement. Therefore, the deficiency caused by lack of transverse reinforcement was not considered. The model was validated against an experimental data set, including only interior connections.

Jeremic and Bao [67] attempted to simulate the panel shear and interface response of reinforced-concrete-beam–column connections by simplifying the model by Lowes and Altoontash [47]. In order to incorporate the flexural distortion at the interface, the authors employed both finite and zero-length linear springs. Tajiri et al. [68] proposed a macro-element for the analysis of RC joints in elasto-plastic frames. In this model, the RC sections were represented by rigid components that remained as a plane before and after deformation, while non-linear axial springs were used for depicting the concrete material, reinforcing bars, bond–slip mechanism, and shear distortion. Bao et al. [69] modified the model by Lowes and Altoontash [47] for the response estimation of interior joints under lateral loading and capturing progressive collapse mechanism RC frame structures. For capturing the bond–slip mechanism, the authors employed a bilinear response envelope, whereas a symmetric multi-linear M-θ relationship was used to capture the joint panel shear based on the MCFT. Anderson et al. [70] developed a joint model based on a hysteretic shear-stress–shear-strain model for deficient RC connections lacking transverse reinforcement utilizing an experimental database comprising a wide range of deformation histories and shear demands for model calibration.

**Figure 11:**
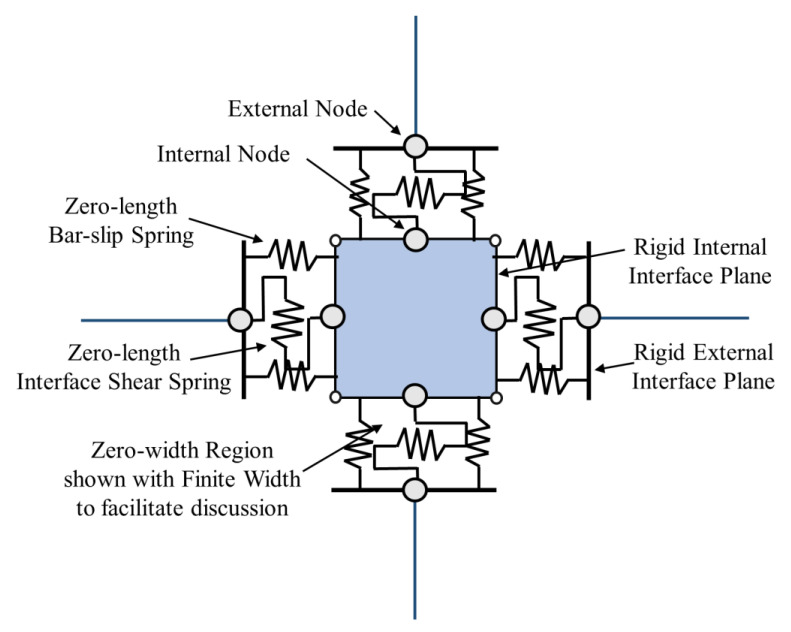
Joint super-element with zero-length interface bar–slip springs placed at the centroid of flexural steel, reproduced from literature [71].

Mitra and Lowes, 2007 [71], modified an existing multi-spring macro-model originally presented by Lowes and Altoontash [47]. The modifications were aimed at improving the level of accuracy and versatility. The applicability was extended to GLD structures, accounting for strength and stiffness degradation. As per the model stress transfer mechanism, the shear stress is transferred through the confined concrete strut and resisted by the compression forces from the connected beams and columns as shown in Figure 11. The model is incorporated in the OpenSees non-linear analysis package. The model was implemented by Pan et al., 2017 [72], in a global frame analysis to study the effects of joint deformations on the wholistic frame response. The model works on the principles of the model previously proposed by Lowes et al., except for the delay in the initiation of the bar–slip mechanism and the use of hysteretic damage instead of an envelope for softening. The strength loss is evaluated using the given equation.

$$\delta_{i}^{f}=\alpha_{1}\left({\tilde{d}}_{max,i}-{\tilde{d}}_{ult}\right)\leq \delta_{lim}^{f}\;for\;{\tilde{d}}_{max,i}>{\tilde{d}}_{ult}$$

$$\alpha_{1}=\frac{\delta_{lim}^{f}}{1-d_{ult}}$$

$${\tilde{d}}_{ult}=\max\left[\frac{d_{ult,comp}}{D_{min}},\;\frac{d_{ult,ten}}{D_{max}}\right]$$

where *d_ul_*_t,*comp*_ and *d_ult_*_,*ten*_ are the ultimate deformation of tension and compression steel, respectively. Minimum and maximum strain capacities are represented by *D_min_* and *D_max_*, respectively. $\delta_{lim}^{f}$ corresponds to the minimum strength being equal to the bond strength.

However, the data used for the calibration and validation of the model included only interior joints. Furthermore, the model is suitable for beam–column joints with minimum reinforcement, and not applicable for joints without transverse reinforcement.

**Figure 12:**
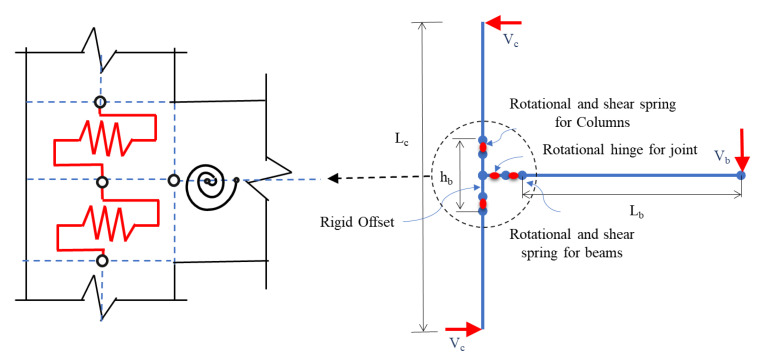
Multi-spring joint modeling philosophy and incorporation of joint and interface shear mechanism, reproduced from literature [73].

Sharma et al. [73] proposed a new modeling approach (Figure 12) to simulate the joint shear response under monotonic loading. The failure criteria are based on the joint’s limiting principal tensile stress. The actual deformations in the sub-assemblage caused by the shear deformation of the joint panel are used to determine spring properties. The hysteretic rule in the study by Takeda et al. [14] was used for cyclic behavior. For joints without a column axial load, the below equations were used for the principal tensile stress and corresponding joint horizontal shear.

$$P_{t}=\frac{\alpha \tau }{2}\left(1-\sqrt{1+\frac{4}{\alpha^{2}}}\right)$$

$$\tau =\frac{2P_{t}}{\alpha \left(1-\sqrt{1+\frac{4}{\alpha^{2}}}\;\right)}$$

For joints with a column axial load, the vertical and horizontal shear response was predicted through the following equations.

$$\sigma =\frac{V_{jv}+P}{b_{c}h_{c}}$$

$$\tau =\frac{V_{jh}}{b_{c}h_{c}}$$

However, the model does not count for the bar–slip mechanism, but considers it indirectly through shear simulation as in the case of Pampanin et al., 2003 [26].

**Figure 13:**
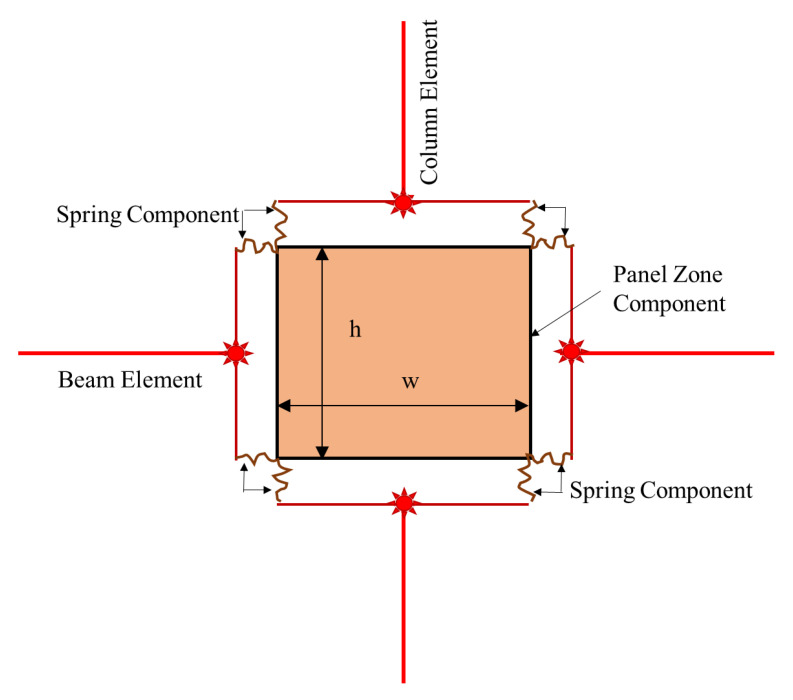
Joint element with eight linear and rotational springs and a central joint panel component to capture interface and panel shear along with bar–slip mechanism, reproduced from literature [74].

Ning et al. [74] presented a new model used to simulate the strength and stiffness degradation for deficient RC connections in subsequent cycles along with the pinching effect during inelastic analysis. The proposed joint element consists of eight linear and rotational springs and a central joint panel component. The joint shear is captured by using a 2D joint panel, whereas the bond–slip mechanism is captured through linear springs at the interface as shown in Figure 13. The model was calibrated/validated at both the component and structural levels with experimental data of deficient RC connection sub-assemblages and lightly reinforced frames. The shear stress and shear strain response of the joint was stated as

$$\tau =\sigma_{sc}\frac{A_{sc}\sin\theta }{h}$$

$$\gamma =\frac{\varepsilon_{sc}}{\sin\theta \cos\theta }$$

where τ is the panel shear stress, $\sigma_{sc}$ represents the stress in the diagonal strut, $A_{sc}$ represents the strut width, θ is the angle of strut, h is the depth of the beam, and $\varepsilon_{sc}$ is the diagonal strain.

However, the model cannot predict the nonlinear response caused by the loss of the shear transfer phenomenon at the interface.

**Figure 14:**
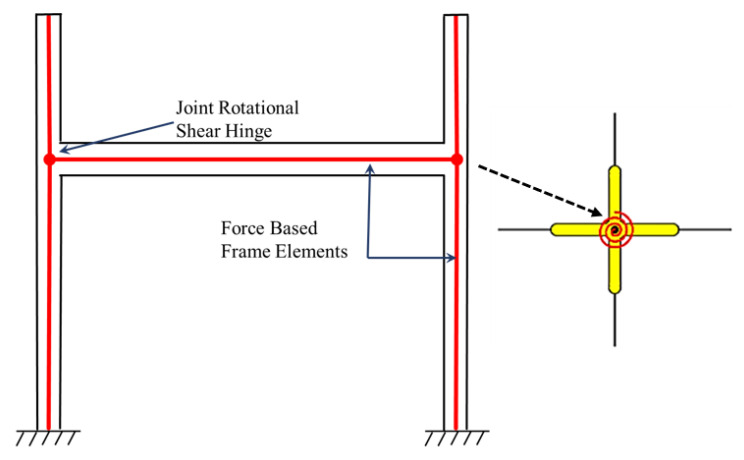
Beam–column joint element comprising zero-length elements for panel shear and lump plasticity hinges for interface shear response, reproduced from literature [8].

Khan et al. [8] proposed a simplistic approach to predict the RC joint response in the non-linear domain. The panel inelastic shear response was simulated through a central zero-length element, whereas the interface shear response was predicted by a lump plasticity hinge as shown in Figure 14. The M-θ constitutive relationship was defined based on the shear stress–strain response provided by Kim and LaFave [37].

$$v_{j}=\alpha_{t}\beta_{t}\eta_{t}\lambda_{t}{\left(JI\right)}^{0.15}{\left(BI\right)}^{0.30}{\left(f_{c}^{\prime }\right)}^{0.75}$$

$$\gamma_{j}=\alpha_{\gamma t}\beta_{\gamma t}\eta_{\gamma t}\lambda_{\gamma t}BI\;{\left(JI\right)}^{0.1}{\left(\frac{v_{j}}{f_{c}^{\prime }}\right)}^{-1.75}$$

where the coefficients α and β represent in-plane and out-of-plane geometries, respectively, and η and λ represent joint eccentricity. *JI* and *BI* are the joint transverse reinforcement index and beam reinforcement index, respectively.

Sivaselvan and Reinhorn’s [75] multi-linear hysteretic rule was used to predict the joint’s cyclic response. In this modeling technique, the moment capacity of the rotational spring was determined using the scissor model presented by Alath and Kunnath, [3] as shown in Figure 7a. However, the model failed to simulate the shear response of RC beam–column connections without transverse reinforcement.

**Figure 15:**
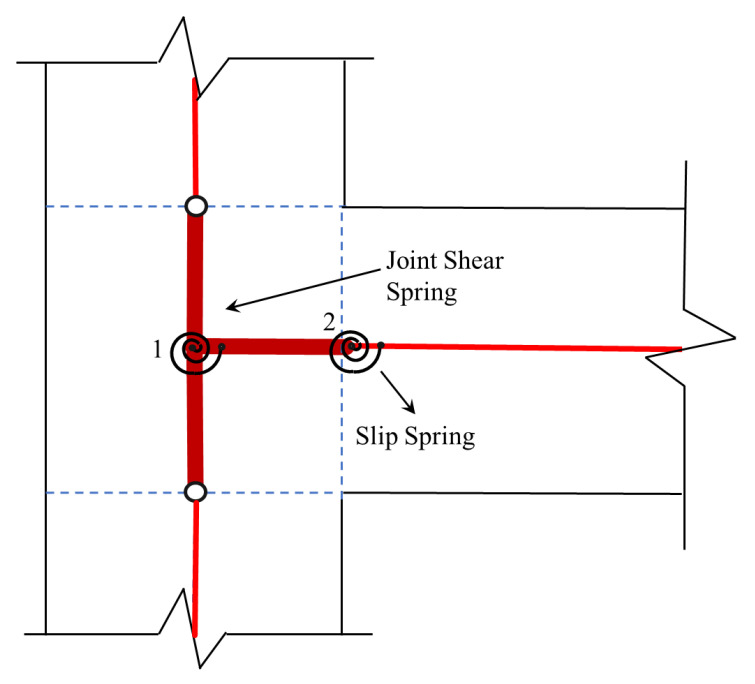
Modified scissor model with two rotational springs in series to demonstrate panel and interface shear failure, reproduced from literature [7].

Grande et al. [7] recently modified the well-known “scissors model” [3] to incorporate the panel shear and bar–slip mechanism. To model the panel shear response and bond–slip mechanism at the interface, the authors introduced two rotational springs in series as shown in Figure 15. For the hysteretic behavior and pinching effect, the “pinching4” uniaxial material model [47], incorporated in OpenSees [76], was used. The load-deformation path was defined by using modified compression field theory (MCFT) [51]. The proposed model provides sufficiently accurate results compared to previous models.

The conversion mechanism from shear stress to moment curvature demand has been explained under summary of Section 1.3.1. The model has been implemented in the nonlinear open-source finite element platform, OpenSees.

Multi-spring models are based on the superposition of joint mechanics with experimental observations and are more accurate. However, in some circumstances, huge amounts of experimental data are required for calibration. The following are some of the models’ drawbacks:

1. They necessitate a greater computational effort than rotating hinge models.
2. They frequently necessitate the inclusion of a unique feature in software.
3. Most available models are incompatible with gravity-designed frame joints.

### 1.4. Lumped-Plasticity Approach

Lumped plasticity models consider localized yielding of the zero-length regions at the member ends, where plastic hinges develop [77,78,79]. The vast acceptance of the lumped plasticity approach in research and the industry is accredited to its exceptional simplicity, computational efficiency, and reasonable precision in simulating the yield and ultimate strength. However, the model cannot estimate deformations corresponding to the yield and ultimate loads with the required accuracy. Furthermore, the strength estimation is highly dependent on plastic hinge locations. The damage level at the ultimate load is often underestimated, as it is also highly sensitive to the plastic hinge location [78].

#### Spread Plasticity Approach

In the spread plasticity approach, plastic hinges are extended over a finite length of the member ends that undergo non-linear deformation [80,81,82,83]. These models can capture the seismic behavior with minimum computational effort. However, they may be subjected to serious limitations, since it does not account for non-linear axial deformations and assumes that plastic hinges develop only due to flexure [83]. The spread plasticity models that account for the junction plasticity only are suitable for the low-rise structure (gravity load < 0.8). The model’s accuracy is reduced when global responses in terms of translation, rotation, and story drift are estimated. A new more accurate spread plasticity model was proposed by Michael Kyakula and Sean Wilkinson [82], which improves the computational accuracy of structural deformations, joint curvature, and lateral story displacement by 25%, 69%, and 55%, respectively, and is sensitive to the applied gravity load and amount of flexural reinforcement.

### 1.5. Finite Element (FE) Numerical Modeling

Another more recent and accurate approach used to model and analyze joint mechanics is finite element (FE) numerical simulations. These models are bifurcated into micro-scale and meso-scale models. In micro-scale modeling, all of the material heterogeneities and interface mechanisms are modeled separately. A meshing technique is used to define finite elements on which the response can be analyzed and predicted. A structural or non-structural mesh can be defined depending on model requirements. The section is also divided into multiple fibers to study its response in terms of the stress distribution and corresponding deformations. As obvious from the above discussion, these models give more precise results but at a higher computational cost, since these are hardly used by practicing engineers and structural designers [84,85,86,87]. Furthermore, such approaches are rarely used for the analysis of entire buildings due to a massive computer memory demand.

## 2. Conclusions

In the past few decades, unprecedented progress has been made in understanding, analyzing, and assessing the response of RC beam–column joints. Multiple modeling techniques have been proposed to capture the non-linear response of joints in RC frames subjected to lateral loads. The non-linear response of RC joints is dominated by two main mechanisms: panel shear deformation and the bar–slip mechanism, which have been modeled using diverse approaches. In the recent past, the modeling approaches have evolved significantly, with an improved accuracy and reduced computational effort. The early models were based on experimental investigations; however, they were proven to be unreliable as they were dependent on extensive experimental data. More elaborate and precise models were proposed as our understanding of the behavior of connections grew.

- In rotational spring models, a central zero-length element is used to connect the elastic beams and columns to the joint. The entire non-linear behavior is lumped in a single rotational spring, due to which, it is difficult to individually assess the joint panel shear, interface shear, and bar–slip mechanism.
  - In this scenario, determining the M-θ properties necessitates extensive experimental data for the calibration and validation of the model.
- A much more realistic and widely accepted approach is the multi-spring modeling technique, as it captures the individual responses using separate springs for each joint mechanism and is applicable to the analysis or assessment of entire structures.
  - The behavior is presented through non-linear translational or rotational springs.
  - With these models, more accurate and realistic simulation results can be obtained with a slight increase in computational effort, and computer memory requirements are compared to lumped plasticity or rotational spring approaches.
  - The computational effort and computer memory demand is still significantly low compared to the FE model.
  - However, a separate material and constitutive model needs to be assigned to each zero-length spring/hinge for governing its non-linear response.
  - Several multi-spring models are not suitable for RC frames without shear reinforcement in the joint core, which is a more critical deficiency in gravity-designed frames.
  - The most accurate approximation is carried out by using the model by Lowes et al., 2007, as it covers almost all of the mechanisms individually through linear and rotational springs. However, the computational effort is significantly increased.
  - The scissors model is the simplest, with minimal computational effort. However, only the joint panel rotation is captured. The bar–slip mechanism at the interface is not considered.
  - In multi-spring models, the model by Grande et al. can be considered as the most efficient in terms of both the accuracy and computational effort. Both the interface and joint panel response have been covered efficiently using two springs. The fact that the bar–slip mechanism is more critical at the beam–joint interface is effectively used to minimize the computational effort.

As a result, there is still room for more realistic and computationally efficient models to be developed that can not only accurately anticipate the behavior of even the most intricate beam–column connections, but can also be implemented in general-purpose nonlinear analysis systems.

## Data Availability

Not applicable.
